# Comparing Three Data Mining Algorithms for Identifying the Associated Risk Factors of Type 2 Diabetes

**DOI:** 10.29252/ibj.22.5.303

**Published:** 2018-09

**Authors:** Habibollah Esmaeily, Maryam Tayefi, Majid Ghayour-Mobarhan, Alireza Amirabadizadeh

**Affiliations:** 1 Department of Biostatistics, School of Health, Mashhad University of Medical Sciences, Mashhad, Iran; 2 Department of Modern Sciences and Technologies, School of Medicine, Mashhad University of Medical Sciences, Mashhad, Iran; 3 Biochemistry of Nutrition Research Center, School of Medicine, Mashhad University of Medical Sciences, Mashhad, Iran; 4 Medical Toxicology and Drug Abuse Research Center (MTDRC), Birjand University of Medical Sciences, South Khorasan, Iran

**Keywords:** Data mining, Diabetes type 2, Support vector machine

## Abstract

**Background:**

Increasing the prevalence of type 2 diabetes has given rise to a global health burden and a concern among health service providers and health administrators. The current study aimed at developing and comparing some statistical models to identify the risk factors associated with type 2 diabetes. In this light, artificial neural network (ANN), support vector machines (SVMs), and multiple logistic regression (MLR) models were applied, using demographic, anthropometric, and biochemical characteristics, on a sample of 9528 individuals from Mashhad City in Iran.

**Methods:**

This study has randomly selected 6654 (70%) cases for training and reserved the remaining 2874 (30%) cases for testing. The three methods were compared with the help of ROC curve.

**Results:**

The prevalence rate of type 2 diabetes was 14% in our population. The ANN model had 78.7% accuracy, 63.1% sensitivity, and 81.2% specificity. Also, the values of these three parameters were 76.8%, 64.5%, and 78.9%, for SVM and 77.7%, 60.1%, and 80.5% for MLR. The area under the ROC curve was 0.71 for ANN, 0.73 for SVM, and 0.70 for MLR.

**Conclusion:**

Our findings showed that ANN performs better than the two models (SVM and MLR) and can be used effectively to identify the associated risk factors of type 2 diabetes.

## INTRODUCTION

Diabetes mellitus (DM) is rapidly growing in prevalence, thus posing a major public health challenge globally. The estimated number of diabetic adults was 382 million in 2013, while the prediction for year 2035 is 592 million[1,2]. Diabetes is associated with health complications such as cardiovascular, renal, and retinal diseases; therefore, it is important to identify individuals having a high risk of diabetes, in order to control other health risks.

Different models have been developed during the last two decades for evaluating the risk of DM, but none of them have been found to achieve the desired accuracy, hence having a limited clinical utility[3,4]. Studies have also suggested an connection between anthropometric factors and DM[5,6].

Data mining involves choosing, analyzing, and modeling huge amounts of information, aiming to reveal undisclosed patterns or correlations that propose obvious and beneficial results[7,8]. This method has progressed fast in the recent years. Several researches have used data mining in order to investigate unknown variables. In addition, some predictive models are created in medicine[9,10].

The multiple logistic regression (MLP) is a nonlinear regression approach to predict a categorical and a dependent variable. This method has been used for identifying the risk factors for various diseases through patients’ history, characteristics, and other risk factors. The logistic model measures the probability of the considered illness y (y = 0 if the participant does not suffer from the illness; if not, y = 1) as a function for the predictive risk factor values. If the person suffers from this illness, the conditional probability can be obtained by p (y = 1|X) = p (X), and the logistic model becomes: log [p (x)/1 = p (x)] = B_0_ + B_1_x_1_ + B_2_x_2_ + B_k_x_k_ in which X = (x_1_, x_2_, …, x_k_) indicates the k’s risk factors vector through the logistic regression model[11,12].

The support vector machines (SVMs) create appropriate boundaries between the information sets through solving a quadratic optimization problem. By making the use of various kernel functions, different amounts of flexibility and nonlinearity can be attached to the model. Since these features could be elicited from high level statistical ideas, and their generalization error bounds can be measured, a great deal of research has been conducted on SVMs during the last years[13,14].

The artificial neural networks (ANNs) are suitable data mining instruments that are utilized for building nonlinear and complex models. The present study utilized a common ANN architecture named multi-layer perception network with back-propagation algorithm, which is surely the most widely used and carefully deliberated ANN architecture[15,16]. The multi-layer perception networks are feed-forward neural networks that are instructed with standard back-propagation algorithms and are considered as a strong function approximate for the problems of classifying and predicting[17].

In public health, it is common to use MLR for identifying factors associated with the disease and for developing predictive models[18,19]. Some data mining techniques have already been applied for medical conditions[9,20,21] and for predicting diabetes[8,22,23]. The present study aims at comparing ANN, SVM, and MLR for identifying associated risk factors of DM.

## MATERIALS AND METHODS

### Experimental sample

A sample of 9528 subjects were enrolled in the Mashhad Stroke and Heart Atherosclerotic Disorders Study at Mashhad University of Medical Sciences (MUMS), Mashhad, Iran[24]. The Ethics Committee of MUMS has approved the protocol, and all the participants have been given an informed written consent.

### Data collection (anthropometric and biochemical measurements)

Demographic characteristics consisted of age, gender, marital status, education, cigarette smoking habit, physical activity level (PAL), family history of diabetes, and depression score. The depression score was evaluated using Beck’s depression inventory II. Anthropometric information included weight, height, waist, and hip circumference. Systolic and diastolic blood pressures were measured as described earlier[12]. Biochemical measurements were composed of fasting blood glucose, fasting serum triglycerides (TGs), total cholesterol (TC), HDL- and LDL-cholesterol, and high-sensitivity C-reactive protein (hs-CRP), as previously described[24].

### Phenotypic definition of type 2 diabetes mellitus

Phenotypic definition of Type 2 DM was specified based on the fasting blood glucose level of 126 mg/dl or higher.

### Artificial neural network

ANN is a data mining tool and is used for constructing non-linear prediction models[7]. A multilayer neural network has three layers, namely an input layer, a hidden layer, and an output layer. Each layer has nodes that are connected by links from one layer to the next. Nodes in the input layer represent predictors, while in the output layer, the nodes are viewed as outcome variables[15]. One of the most applications of neural network is multilayer back-propagation learning algorithm that has ability of modeling a non-linear systems[25]. Interpretation of neural networks is more complicated than other statistical models; however, the neural network is used in different medical fields[25,26]. The structure of perceptron network is composed of some nodes with an activation function that are held in different layers. Each node by its weight coefficients collects the results of all previous nodes and converts it to next layer through activation function. The number of nodes in each layer of network depends on the structure of the investigated subject[15,25]. In a perceptron network with a hidden layer, the amount of i-th output is calculated using the following formula in which n: the number of observations, M: the number of hidden layer nodes, p: the number of entrance layer nodes, w_js_: weight related to χ_is_ enter in i-th node, χ_is_: weight of i-th node, b_0_ and b_j0_: bias of middle and output layers, respectively, Ø_2_ and Ø_1_: activation functions of hidden and output layers.


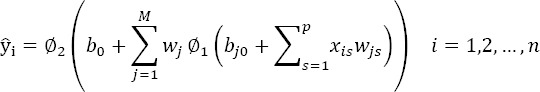


The activation function of hidden layer usually is non-linear (hyperbolic tangent or sigmoid), and conversion function of output layer can be linear or non-linear[15,27].

The aim of ANN is calculating the proper weight for network. One way of measuring the weights is back-propagation algorithm. Back-propagation rule consists of two paths. One path is forward path, in which entrance vector applies to perceptron network, and its effect expands into output layer through a middle layer. The constructed output vector in the output layer makes the real response of the perceptron network, which is named as the backward path, in which the parameters of network are considered fixed. In this path, in contrast to the going path, the parameters of perceptron network are changed and adjusted. This adjustment is done according to the error correction law. These paths are repeated until the parameter estimates (bias and weight) are adjusted. The process of measuring the proper weight is considered as the learning process. For doing this process, weight coefficients will be changed to reduce the goal function of network, which is considered as mean square error[28,29].

### Support vector machine

SVM is a data mining tool and is a supervised classification technique. This method can be used for prediction when the outcome variable is binary. SVM constructs multi-dimensional hyper-planes separating the two classes while maximizing the margin between the two classes. SVM uses kernel functions and has the ability to discriminate between classes that are not linearly separable[30]. The dataset is divided into two sets with the training dataset comprising of 6654 cases (70%) and testing dataset containing 2874 cases (30%). Each model is developed using the training dataset and tested using the testing dataset[31]. In case of each model, the incidence of type 2 diabetes is predicted, and confusion matrix is constructed in order to measure the accuracy of the model. Accuracy is measured as the proportion of cases classified correctly. Sensitivity is measured by the proportion of positive cases classified correctly, while specificity is determined by the proportion of negative cases classified correctly[7]. Mathematically, if TP stands for true positive, TN for true negative, FP for false positive, and FN for false negative, then accuracy = (TP + TN)/(TP + FP + TN + FN), sensitivity = TP/(TP + FN), and specificity = TN/(FP + TN).

### Statistical analysis

The data were analyzed using R version 3.0.2. All variables were analyzed to generate descriptive statistics, chi-square tests, independent sample *t*-tests for variables with a normally distributed variables, and Mann-Whitney tests for non-normally distributed variables. MLR was used to identify factors that are strongly associated with type 2 DM.

## RESULTS

### Characteristics of the population

Descriptive statistics for anthropometric and biochemical characteristics are reported in Tables 1 and 2, respectively. The number of subjects having type 2 diabetes was 1361, which were selected from 9528 subjects. The mean age of diabetic individuals was higher than non-diabetic individuals (52.01 ± 7.2 vs. 47.70 ± 8.1). Of 1361 diabetic individuals, 843 (61.9%) were female, 1239 (91%) were married, and 783 (57.5%) were unemployed. Subjects having DM were significantly (*p* < 0.05) older and had higher BMI, systolic blood pressure, diastolic blood pressure, serum TC, and LDL cholesterol, as well as a lower level of HDL-C, compared to the individuals without DM (Table 1).

**Table 1:** Comparison of baseline characteristics between diabetes and non-diabetes groups

Variables	Diabetes (n =1361)	Non-diabetes (n = 8167)	*p* value
Age (year)	52.01 ± 7.2	47.70 ± 8.1	<0.001
BMI (kg/m^2^)	27.76 ± 4.7	28.78 ± 4.4	<0.001
Gender			
Male	3277 (40.1%)	518 (38.1%)	=0.04
Female	4890 (59.9%)	843 (61.9%)	
Marriage status			
Single	54 (0.7%)	5 (0.4%)	<0.001
Married	7636 (93.5%)	1239 (91%)	
Divorced	111 (1.4%)	21 (1.5%)	
Widow	366 (4.5%)	96 (7.1%)	
Education Level			
Low	4319 (52.9%)	878 (64.5%)	<0.001
Moderate	2912 (35.7%)	374 (27.5%)	
High	936 (11.5%)	109 (8.0%)	
Occupation status			
Employment	3125 (38.3%)	400 (29.4%)	<0.001
Unemployment	4283 (52.4%)	783 (57.5%)	
Retired	759 (9.3%)	178 (13.1%)	
Smoking status			
Yes	1775 (21.7%)	272 (20.0%)	=0.05
No	6392 (78.3%)	1089 (80.0%)	
Family history of diabetes			
Yes	1994 (24.4%)	647 (47.5%)	<0.001
No	6173 (75.6%)	714 (52.5%)	
Depression			
Yes	2226 (27.3%)	461 (33.9%)	=0.001
No	5941 (72.7%)	900 (66.1%)	
SBP (mmHg)	121.14 ± 18.2	128.81 ± 18.4	<0.001
DBP (mmHg)	78.91 ± 11.1	81.36 ± 10.4	<0.001
Cholesterol (mg/dL)	189.69 ± 37.8	201.46 ± 46.3	<0.001
LDL (mg/dL)	116.74 ± 34.6	120.49 ± 39.1	<0.001
HDL (mg/dL)	42.73 ± 9.9	42.81 ± 9.6	=0.004
PAL (h per week)	1.59 ± 0.86	1.60 ± 0.64	=0.04
hs-CRP (mg/l)	1.56 [0.95-3.23]*	2.7 [1.3-4.9]*	<0.001
TG (mg/dL)	117 [82-165]*	160 [105-222]*	<0.001

* Median (IQR)

**Table 2:** Multiple logistic regression analysis on the influential factors of type 2 diabetes in training dataset

Variables	B (SE)	OR (95% CI)	*P* value
Age(year)	0.05 (0.004)	1.05 (1.04-1.06)	<0.001
Education			
High		Reference	=0.27
Moderate	0.12 (0.11)	1.13 (0.91-1.39)	=0.59
Low	-0.04 (0.07)	0.96 (0.84-1.11)	
Occupation			
Unemployment		Reference	
Retired	0.16 (0.11)	1.18 (0.94-1.47)	=0.15
Employment	0.46 (0.12)	1.59 (1.27-1.99)	<0.001
Married status			
Divorced		Reference	
Married	-0.22 (0.24)	0.80 (0.50-1.28)	=0.36
Single	-0.67 (0.54)	0.51 (0.17-1.48)	=0.21
Widow	-0.23 (0.26)	0.79 (0.47-1.33)	=0.37
Smoking status			
No		Reference	
Yes	-0.11 (0.07)	0.89 (0.77-1.03)	=0.13
Family history of diabetes			
No		Reference	
Yes	1.03 (0.06)	2.81 (2.5-3.16)	<0.001
Depression			
No		Reference	
Yes		0.76 (0.61-0.95)	<0.001
BMI (kg/m^2^)	0.02 (0.007)	1.02 (1.01-1.02)	<0.001
SBP (mmHg)	0.01 (0.002)	1.02 (1.01-1.02)	<0.001
DBP (mmHg)	-0.01 (0.004)	0.99 (0.98-0.99)	=0.009
LDL (mg/dL)	-0.003 (0.002)	0.99 (0.99-1.001)	=0.1
HDL (mg/dL)	-0.003 (0.004)	0.99 (0.99-1.005)	=0.47
Cholesterol (mg/dL)	0.005 (0.002)	1.005 (1.001-1.008)	=0.007
hs-CRP (mg/l)	0.02 (0.003)	1.02 (1.01-1.02)	<0.001
TG (mg/dL)	0.003 (0.0001)	1.003 (1.002-1.004)	<0.001
PAL (h per week)	0.001 (0.0001)	1.001 (1.001-1.003)	=0.001

SE, standard error

### Multiple logistic regression model

**Fig. 1:**
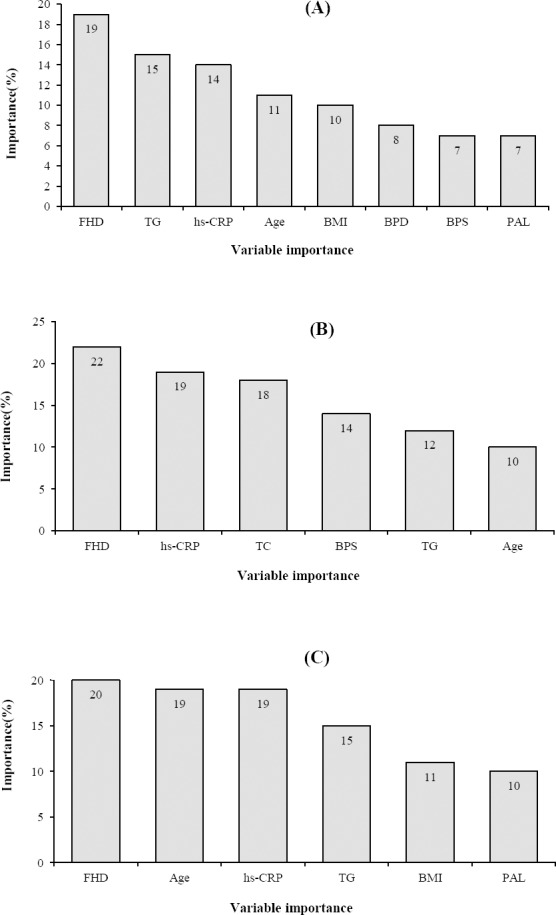
The importance of input variables in MLR (A), ANN (B), and SVM (C) models.

MLR identified a strong association between diabetes and the following characteristics: age, family history of diabetes, BMI, systolic blood pressure, diastolic blood pressure, hs-CRP, TG, and PAL. (Fig. 1A) The ORs of age (95% CI: 1.04-1.06, *p* < 0.001), family history of diabetes (95% CI: 2.5-3.16, *p* < 0.001), and hs-CRP (95% CI: 1.01-1.02, *p* < 0.001) were 1.05, 2.81, 1.02, respectively. The results showed that for one unit of increase in hs-CRP, the chance for diabetes is 1.02 units. The results are reported in Table 2.

### Results of artificial neural network model

For designing the neural network and finding the best model, we performed the test on the least square error function. At the beginning, the learning rate and the number of neurons were considered as 0.05 and 10, respectively. Sigmoid activation function was used in a hidden layer, and linear activation function was used in the output layer. Gradually, the learning rate was increased up to 0.5, and the number of neurons up to 25 increased. All the combinations of different learning rates and the number of neurons were tested.

Subsequently, according to the mean square error, the best model was selected. After considering different combinations, the three-layer perceptron network with 24 nodes in entrance layer, 20 nodes in hidden layer, and 2 nodes in output layer with the rate learning of 0.2 were used, which in comparison with other combinations had the least mean square error (Table 3). The order of important variables in the ANN model containing six parameters was as follow: family history of diabetes, hs-CRP, TC, BPS, TG, and age risk factor diabetic (Fig. 1B).

**Table 3:** The mean predicted error according to the number of hidden layer nodes by neural network

	Number of hidden layer nodes						Learning rate

	20	18	16	14	12	10	
Mean square error	27.04	27.12	27.52	27.56	27.74	28.51	0.05
	26.84	26.94	27.56	27.44	27.80	28.46	0.01
	26.8	27.23	27.39	27.54	27.87	28.38	0.20
	26.94	27.37	27.38	27.51	27.86	28.37	0.50

### Results of support vector machines model

**Fig. 2:**
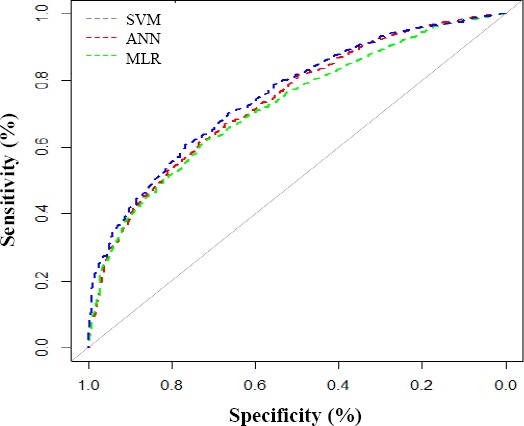
ROC curves of the SVM, ANN, and MLR models in testing dataset.

SVM applied the same 17 characteristics used by ANN and tried sigmoid as well as polynomial kernel functions to identify the best kernel function. Based on the results of the SVM model, family history of diabetes, age, hs-CRP, TG, BMI, and PAL were the most important risk factors related to type 2 diabetes (Fig. 1C). Table 4 summarizes the results on accuracy, sensitivity, specificity, and area under the ROC curve for these three models. As shown in the Table, SVM model had the best performance. ROC curve for the three models is displayed in Figure 2.

**Table 4:** The performance of three models for identifying associated risk factors of type 2 diabetes

Model (%)	ANN (95% CI)	MLR (95% CI)	SVM (95% CI)
Sensitivity	63.1 (59.8-67.5)	60.1 (58.4-63.1)	64.5 (59.8-66.4)
Specificity	81.2 (78.4-84.6)	80.5 (76.4-85.3)	78.9 (76.4-81.7)
Accuracy	78.7 (73.5-82.6)	77.7 (73.5-80.9)	76.8 (73.5-80.9)
AUC	71.5 (68.0-75.9)	70.4 (68.6-73.9)	73.1 (69.2-77.6)

AUC, the area under the ROC curve

## DISCUSSION

In the present study, we developed and explored the effectiveness of three models of data mining, including ANN, MLR, and SVM, in order to identify some potential factors associated with type 2 diabetes in a large population consisted of 9582 subjects with and without diabetes. In line with the previous studies, our results indicated that ANN is better than MLR and SVM for identifying associated risk factors of type 2 diabetes[10,19]

MLR, ANN, and SVM were applied on training data set, and all models were used for the evaluation of testing data set. Based on demographic and biochemical markers, our findings showed that the ANN model had a higher predictive accuracy in comparison to MLR and SVM models.

The sensitivity and specificity are two important factors for the validity of a model[32]; therefore, these two criteria were calculated for the three models. The results revealed that ANN model had more sensitivity and specificity than MLR model and less sensitivity than SVM, which are in agreement with the findings of Meng *et al*.[8] and Sedehi *et al*.[29] studies. Investigations have also suggested that ANN could give a better prediction value than MLR[8,17]. Those studies have compared ANN and MLR models using demographic and anthropometric variables, but we have used biochemical parameters. Moreover, we found that in the ANN model, the family history of diabetes and hs-CRP had a more important role in the identification of individuals with type 2 diabetes, while in MLR model, family history of diabetes, TG and hs-CRP and in SVM model, the family history of diabetes, age, and hs-CRP play a key role in determining diabetes risk, showing that hs-CRP is an important and a common factor in three models for determining the individuals with type 2 diabetes A major strength of the present study is that it was performed in a large number of samples, thus providing a new insight to the application of ANN, MLR, and SVM for investigating new potential risk factors associated with type 2 diabetes in a representative sample of the Iranian population. However, future studies based on cohort studies are needed to give better estimates of the accuracy, sensitivity, specificity, and area under the ROC curve for ANN.

Although we determined and identified the risk factors associated with type 2 diabetes through ANN, SVM, and MLR models using demographic, anthropometric, and biochemical factors of a large population, more investigations are required to assess the clinical applicability of these three models for evaluation of new risk factors of type 2 diabetes.
